# BMC Biophysics reviewer acknowledgement 2015

**DOI:** 10.1186/s13628-016-0028-z

**Published:** 2016-02-09

**Authors:** Julia Simundza

**Affiliations:** BioMed Central, 233 Spring Street, New York, NY 10013-1578 USA

## Abstract

The editors of *BMC Biophysics* would like to thank all our reviewers who have contributed to the journal in Volume 8 (2015).

**Nadav Amdursky**

UK

**Nurit Ashkenasy**

Israel

**Marc Baaden**

France

**Andrew Battle**

Australia

**Timo Betz**

Germany

**Roland Brandt**

Germany

**Robert Brewster**

USA

**Oscar Domenech**

Spain

**Alexander Dunn**

USA

**Paolo Facci**

Italy

**Reza Farhadifar**

USA

**Klemens Fellner**

Austria

**Nir Gov**

Israel

**Ingo Greger**

UK

**Chen Hu**

China

**Mohan Joshi**

USA

**Pavel Jungwirth**

Czech Republic

**Werner Kremer**

Germany

**Douglas Laurents**

Spain

**Timothy Lezon**

USA

**Benzhuo Lu**

China

**Alexander Mackerell**

USA

**Agostino Migliore**

USA

**Namiko Mitarai**

Denmark

**Sorin Mitran**

USA

**Anders Olofsson**

Sweden

**Vytautas Petrauskas**

Lithuania

**Miquel Pons**

Spain

**Mathis Riehle**

UK

**Arun Sampathkumar**

USA

**Gudrun Schappacher-Tilp**

Austria

**Helmut Schiessel**

Netherlands

**Michael Schlierf**

Germany

**Terence Strick**

France

**Piotr Szymczak**

Poland

**Joanna Trylska**

Poland

**M. Ubbink**

Netherlands

**Stephan Uphoff**

UK

**Ady Vaknin**

Israel

**Sivanandam Veeramuthu**

USA

**Chung Wong**

USA

